# Muscle impairments in osteogenesis imperfecta: a narrative review

**DOI:** 10.1093/jbmrpl/ziaf099

**Published:** 2025-06-05

**Authors:** Alex Ireland, Giorgio Orlando, Marie Coussens, Patrick Calders, Hanna Taipaleenmäki, Eric Hesse, Muhammad Kassim Javaid

**Affiliations:** Department of Life Sciences, Manchester Metropolitan University, Manchester, M1 5GD, United Kingdom; Department of Sport and Exercise Sciences, Manchester Metropolitan University, Manchester, M1 7EL, United Kingdom; Department of Rehabilitation Sciences and Physiotherapy, Ghent University, Ghent, 9000, Belgium; Department of Rehabilitation Sciences and Physiotherapy, Ghent University, Ghent, 9000, Belgium; Institute of Musculoskeletal Medicine, LMU University Hospital, LMU Munich, Munich, D-80539, Germany; Musculoskeletal University Center Munich, LMU University Hospital, LMU Munich, Munich, D-80539, Germany; Institute of Musculoskeletal Medicine, LMU University Hospital, LMU Munich, Munich, D-80539, Germany; Musculoskeletal University Center Munich, LMU University Hospital, LMU Munich, Munich, D-80539, Germany; Oxford NIHR Musculoskeletal Biomedical Research Unit, Nuffield Department of Orthopaedics, Rheumatology and Musculoskeletal Sciences, University of Oxford, Oxford, OX3 7HE, United Kingdom

**Keywords:** physical function, sarcopenia, brittle bone disease, physical activity, dynapenia

## Abstract

The aim of this review is to provide an overview of the available evidence on the effects of OI on skeletal muscle. This encompasses multiple components of muscle function, underlying biological and environmental factors, clinical and functional consequences, and relevant epidemiology and therapeutic options. OI is a rare connective tissue disorder causing bone fragility and skeletal deformity, and extraskeletal features, including cardiac and dental abnormalities and hearing loss. The condition is also characterized by pronounced deficits in multiple aspects of skeletal muscle function, including lower muscle strength and power, impaired balance, and greater fatigability, resulting from lower muscle mass and poor muscle quality. These deficits have important implications for multiple aspects of health and general function, including mobility, fall and fracture risk, and the ability to carry out activities of daily living. The muscle weakness and impaired function in OI appear multi-factorial in origin, and factors including deficits in sensory, ventilatory, and metabolic function may compound those observed in muscle mass and quality. Little is known about the epidemiology of muscle in OI, with the exception that more severe OI types are associated with greater impairments in function and mass. Consideration should be given to which aspects of muscle health and function are most relevant for individuals with different OI types. There is a limited evidence base for interventions to improve muscle in OI, and current findings from physical activity and pharmacological therapies are mixed. Muscle represents an important and under-researched area of health and function in OI.

## Introduction

OI is a genetic disorder affecting connective tissue, with an estimated prevalence of 1 in 10 000.^1^ Around 80%-85% of OI cases result from defects in the COL1A1 and COL1A2 genes encoding type I collagen. OI is further subclassified into a series of types based on its genetic basis or the severity of the disorder.^2^ However, it could be considered a continuum with substantial intra- as well as inter-type variation. OI Types I-IV are typically associated with COL1A1 and COL1A2 mutations, although other mutations have been identified for a minority of cases in types II-IV^3^ and for rarer OI types. The phenotype can vary from “mild” type I, where increased fracture risk is common but growth restriction and deformity are absent or mild, to a perinatal lethal type II. For type III, pronounced short stature and moderate or severe bone deformity are typical, whereas type IV is characterized by milder deformity and less pronounced stature deficits.

Genetic analyses have revealed no less than 22 OI types in total to date, affecting processes, including bone mineralization, collagen modification, folding, cross-linking, and the function and differentiation of osteoblasts. The primary clinical consequences of the disorder relate to skeletal health, including low bone mass and fragility, increased fracture risk, and skeletal deformity, including impaired growth. The presence of type I collagen in multiple tissues means that OI also affects several other aspects of health. These include cardiovascular and respiratory function, dental development, hearing, and muscle function, such that OI is considered a connective tissue disorder rather than purely a skeletal disorder.

The aim of this review is to provide an overview of the available evidence on the effects of OI on skeletal muscle. This encompasses multiple components of muscle function, the biological and environmental factors underlying them, their clinical and functional consequences, and relevant epidemiology and therapeutic options. Skeletal muscle (hereafter “muscle”) is essential for almost every life process, from respiration and digestion, to communication, reproduction, and movement while also playing a key role in whole-body metabolism.

This review will examine the intrinsic differences in skeletal muscle function, underlying structure and composition in OI, and contributions from other body systems on which muscle function depends. Sensory inputs are integrated to plan movements, enacted through the nervous system via electrical potentials to the effectors (muscle), with forces then transmitted via tendons. This system is fueled by aerobic and anaerobic energy pathways, in turn dependent on multiple organ function. Even everyday tasks, such as walking are highly complex, requiring muscles to produce large forces, to precisely co-ordinate simultaneous contractions of multiple muscles across different joints, and to sustain repeated movements without undue fatigue. Therefore, the effects of OI on other systems may have secondary consequences for muscle function. Due to the multi-factorial basis of muscle function, and our interest in discussing underlying mechanisms through to epidemiology and intervention, we felt that a narrative rather than a systematic or scoping review was the most effective way to present this information.

## Study selection

A detailed search of PubMed, Scopus, and Web of Science databases was conducted from their inception to July 2024. The following key terms were used: “osteogenesis imperfecta” or “brittle bone disease” AND “muscle” or “strength” or “balance” or “power” or “lean mass” or “hand grip” or “mobility” or “gait.” The literature search was conducted mainly by the corresponding author of this study (AI), with the co-authors vetting or/and providing additional suggestions and/or missed literature when necessary. Where possible, we focused on data on muscle function, structure, and composition in humans with this knowledge complemented by insights from several animal models of OI. The latter have been reviewed in more detail in a recent narrative review focused on extraskeletal manifestations in OI mouse models.^4^ Therefore, the inclusion criteria were publications containing a patient diagnosis or animal model of OI and the characterization of muscle structure, quantity, or function.

## Muscle function in OI

**Figure 1:**
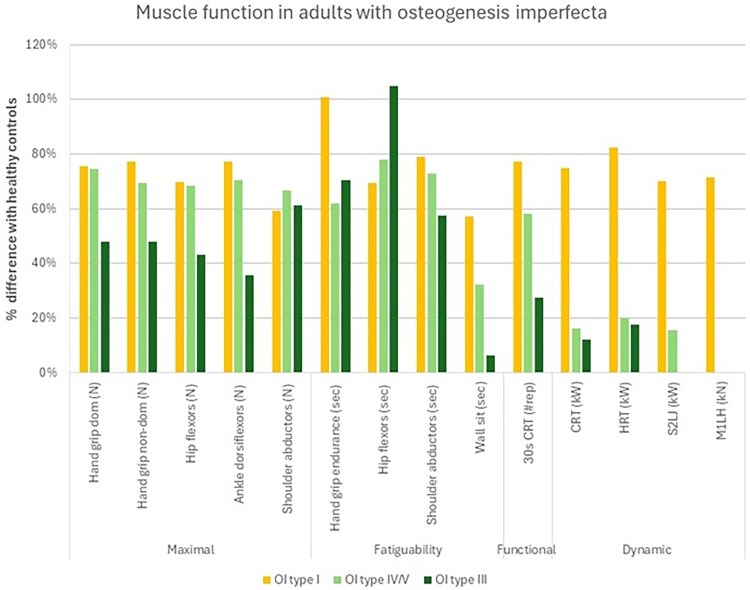
Deficits in different components of muscle function in controls and adults with different OI types—data adapted from Coussens et al.^5^ Abbreviations: CRT, chair-rise test; HRT, heel-rise test; M1LH, one-legged hopping power; S2LJ, countermovement jump power.

Current evidence suggests large deficits in multiple clinically relevant aspects of muscle function in OI (Figure 1). These differences appear independent of differences in body size and muscle mass in both children and adults with OI, particularly for muscle function in dynamic tasks, such as jumping and hopping.^6^^,^^7^ Occasionally, muscle weakness has even been the presenting symptom of OI.^8^ At a fundamental level, muscle strength (ie, the maximal capacity to produce force) across multiple upper and lower limb muscle groups is lower in adults^5^ and children^9–11^ with OI compared with age-matched healthy individuals in both static and dynamic tasks, with a tendency for greater deficits in more severe OI types. For example, the 25% (types I, IV, and V) and 52% (type III) lower hand grip strength observed in adults.^5^ These findings mirror those in mouse models of OI, with larger deficits in electrically stimulated tetanic force in more severe models.^12–16^

**Figure 2:**
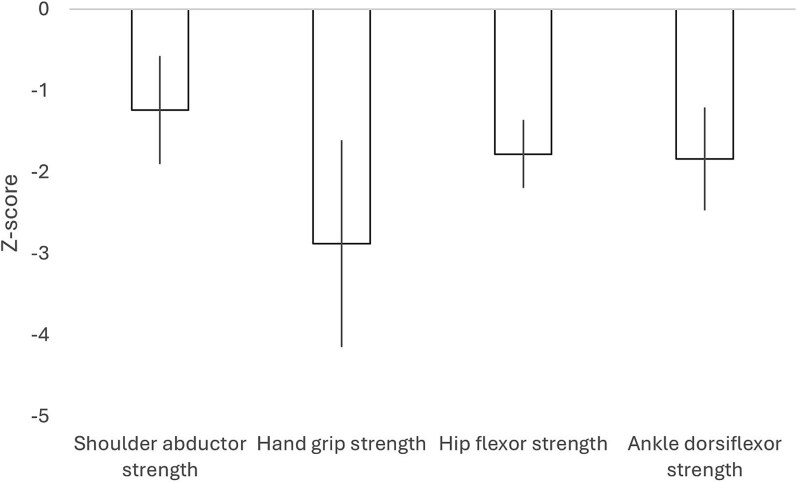
Deficits in isometric strength at multiple joints in children (mean age 13.3 ± 3.9 yr) with OI type I relative to normative age and sex-matched data. Data adapted from Takken et al.^9^ and presented as mean and 95% CI.

Only 2 studies have examined objective quantitative measures of isometric that is static muscle strength across multiple muscle groups. Across multiple muscle groups, mean deficits in adults (Figure 1)^5^ and children (Figure 2)^9^ with type I were relatively similar (hand grip strength 25% lower in adults and 36% lower in children than controls, hip flexors 30% and 32%, ankle dorsiflexors 23% and 30%, and shoulder abductors 41% and 26%). The magnitude of deficits was also similar across muscle groups in individuals with OI types III and IV assessed in the adult study only. Data from children and adolescents with type IV suggests that there may be relative preservation of upper limb function in individuals with reduced mobility, due to the use of wheelchairs or walkers.^17^

Bone mass is regulated in response to the peak forces the skeleton experiences,^18^ with the largest forces produced by muscle.^19^ As a result, within the general population strong positive associations between muscle size (as a proxy for function) and bone mass and other strength indicators are evident.^20^ While muscle size is a strong predictor of maximal strength or power, it is important to note that a muscle produces much lower forces when shortening rapidly, such as the quadriceps during take-off for a vertical jump, than when lengthening rapidly, such as the same muscles on landing from the jump.^21^ As a result, the limitations of muscle mass as an indicator of muscle influence on bone have been demonstrated, for example, by different muscle–bone relationships in the 2 arms of tennis players.^22^ Given these mechanical muscle–bone relationships, in addition to intrinsic deficits in bone mass and quality in OI, lower muscle strength likely makes a substantial contribution to bone weakness and in turn fracture risk in this population.^6^^,^^7^ However, the precise mechanisms and whether they differ from those in individuals without OI remain unclear.

Children with OI type I had similar muscle–bone relationships to control children^7^ in a study, where bone was examined at 4% and 14% distal-proximal tibia sites,^7^ where bone strength against the mainly compressive loading experienced is highly dependent on bone mass. Muscle function was assessed using single-legged hopping involving high-speed eccentric (muscle lengthening) contractions under which high peak forces are produced. In a study in adults with OI, the muscle–bone relationships (although highly significant) differed from controls^6^ similar to observations in a homozygous *oim* mouse model.^15^ Homozygous *oim* mice model severe human type III OI, which is characterized by bone fragility, reduced BMD, and smaller body size.^23^ Despite lower peak force, *oim* muscles were still capable of creating osteogenic levels of strain albeit at much higher percentages of maximum force. The higher bone tissue level stiffness observed in OI^24^ may contribute to these observed muscle–bone differences, as a higher level of force would be required to engender the same level of strain. However, it is unclear why this would affect only adults when material quality is also altered in children with OI.^25^

An alternative explanation for the differing results in the two muscle–bone studies could be methodological. In adults, bone was assessed at the tibia mid-shaft, where bending and torsional strains are important and for which bone geometry also contributes to performance. Muscle function was assessed via maximal hand grip (isometric contraction), and peak counter-movement jump take-off power (high-speed concentric contraction), and these various lower and upper limb measures are only weakly related.^26^ Comparison of muscle–bone relationships using equivalent methodologies is required to understand whether and how muscle–bone relationships differ between adults and children with OI. These previous studies in children^7^ (OI *n* = 20, control *n* = 30) and adults^6^ (*n* = 27 for both groups) of both sexes and different ages may also have been underpowered to detect muscle–bone group interactions that can require large study numbers.

Muscle power (ie, the combination of force and velocity) is the another component of muscle dysfunction in OI, which contributes to impaired mobility,^27^ as well as having implications for fall arrest. Deficits in muscle power have been reported at the lower limbs during tasks, such as hopping and jumping, in individuals with different OI types and of all ages,^5^^,^^28^ as strength deficits are compounded by a reduced rate of force development.^7^ Although these dynamic tasks have not been performed in animals, reduced rate of torque development and peak torque in *oim* mice are in line with this suggestion.^15^ This results in larger deficits than those observed in force alone and could also explain larger deficits in more severe OI types and in adults than children. For example, countermovement peak power mean values 30% (type I) and 85% (types IV and V) lower in adults^5^ and mean values 50% lower (type IV) in children, although deficits in type I children were modest (6%).^17^^,^^28^ Muscle power has not been assessed in the upper limbs in individuals with OI.

Motor control is also affected, with poorer balance performance in children with OI type I.^29^^,^^30^ This component of muscle function has not been assessed in adults with the condition or more severe OI types, although one animal study found impaired motor control and balance in a non-collagenous *Crtap−/−* OI model.^31^ Given bone fragility inherent in OI, this has important implications for fall risk, although surprisingly, fall incidence does not appear to have been measured systematically in OI groups of any age. Motor control deficits emerge at a very early age, with delayed motor development evident across OI types with walking onset 3 mo later in type I and 33 mo later in type III than controls.^32^ Given the importance of motor development for lifelong bone health,^33–35^ this may also contribute to bone weakness and fracture risk in OI.

In addition to motor control, muscle fatigability (ie, the ability to perform repeated or sustained movements without substantial reductions in strength or power) is also impaired in OI. Adults with different OI types completed fewer repetitions in a 30-s chair rise test and in several static endurance tests involving upper and lower body muscle groups (Figure 1), although there was little evidence of differences between OI types and upper or lower limb performance.^5^ While hand grip fatigability in these individuals was similar to controls, the test is relative to the individual’s maximal grip so lower values in OI would mean a lower workload during the test. *Oim* mice display a decay in tension during sustained tetanic contractions, which indicates a defect in calcium handling, and hence an intrinsic inability of the muscle fiber to generate sustained force.^13^ Muscle fatigability is more pronounced in severe (*oim/oim*) animal models with mixed results in milder phenotypes (*+/G610C* and *+/oim*).^13^^,^^36^ This component of muscle function has not yet been assessed against controls in children.

**Figure 3:**
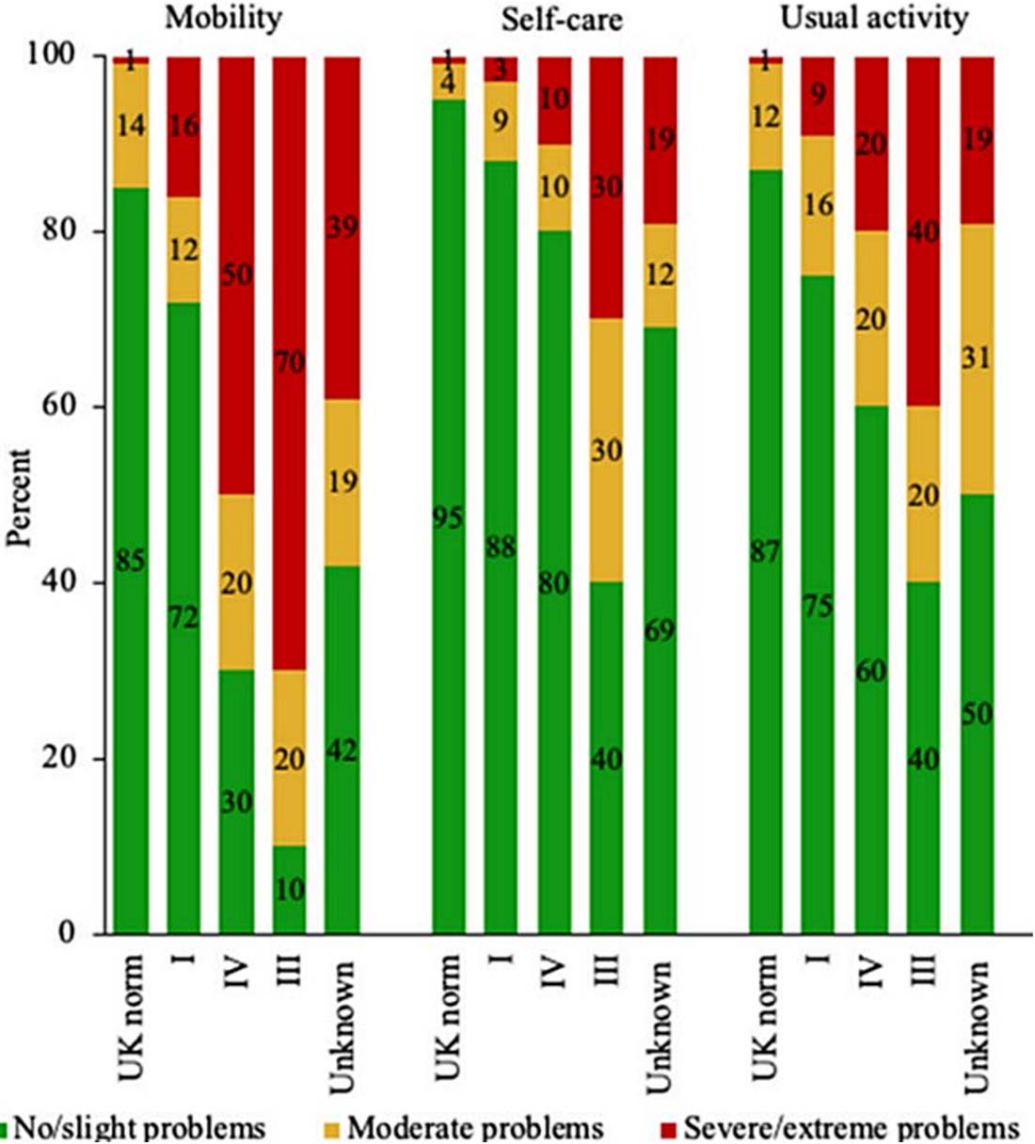
Components of physical function assessed by EQ-5D-5L questionnaire in adults with OI types I, III, IV and unknown type relative to UK population normative values.^27^

The implications of these deficits are evident in higher levels of systemic fatigue and lower levels of physical function, including self-care and mobility in OI (Figure 3).^27^^,^^37–39^ Adolescents with OI type I have 17% lower cycle exercise capacity indicated by lower VO_2_ peak,^9^ and approximately 50% lower cycle peak power^40^ compared to control data from the same authors.^41^ In contrast to type-independent findings in isolated muscle assessments, functional capacity assessed by a low-intensity 6-min walk test was 30% lower in OI type I adults, and over 60% lower in the minority of type III individuals able to perform the test.^32^

More pronounced deficits in these tests may reflect the greater contribution of cardiorespiratory function to performance than in isolated muscle tasks. Also, that muscle weakness means that individuals with OI are working at a greater percentage of their peak work capacity. The reduced capacity of muscle and tendon collagen to store and release energy during movement in OI may also be a relevant factor in locomotory assessments. While this has not been explored directly, children and adolescents with type I OI had greater deficits in peak hop force (which is highly dependent on the ability to store and release elastic energy) than in peak jump power.^28^ The same pattern was not seen in adults with type I, in which relative performance in the two tests was similar.^5^ This suggests differential patterns of aging in OI from controls, in which jump power but not hop force is much lower in older age.^42^^,^^43^

Altered muscle properties can combine to affect whole-body movement in everyday tasks. Slower gait speed in children with OI results from altered kinetics (forces used to move the body) and kinematics (joint and limb angles).^44–46^ In turn, this could result from reduced muscle strength and power, or alternatively a purposeful strategy to avoid excessive loading of vulnerable structures.^47^ Joint hypermobility, reduced joint range of motion and a higher number of fractures and surgeries, particularly evident in more severe OI types, also appear to limit whole-body movement^11^^,^^48^ while foot orthoses may alleviate these gait problems.^49^

## Sensorimotor deficits

The starting point of movement is the sensory information used to inform development of appropriate motor responses. The vestibular system detects movement and orientation of our head in 3D space and provides important information for movement including balance. A study of adults with OI types I-IV found a high level of vestibular dysfunction with over 50% of participants reporting vertigo^50^—no comparison was made between different OI types. This impairment may relate to mechanical nerve damage caused by skull base abnormalities. Deficits in hearing and vision^51^ evident in OI may also impact movement planning, although this has not been explored. Similarly, while proprioception (ie, the ability to perceive limb position and orientation in space) is dependent on collagen type I-rich tendon and muscle, and deficits in proprioception have been recorded in other connective tissue disorders,^52^ it remains unknown in OI. Altered skin properties may affect tactile sensitivity relevant to movement, as evident in the *Col1a1^Jrt/+^* mouse model of severe OI.^53^ These mice demonstrated increased sensitivity to heat, cold, and mechanical stimuli, which were not explained by immunocytochemical analyses of relevant tissues, with authors suggesting that hypersensitivity resulted from chronic pain. Impaired cognition is not a feature of OI, nor are neuropathies of the motor system—hence, it seems that central processing and muscle recruitment are unlikely to be affected. However, that balance performance is impaired more by removal of visual information in children with OI type I than controls is supportive of impaired sensory processing relevant to movement.^29^ Also, the only study to examine electrical activity of the muscles in OI found small differences in activation during mastication,^54^ but locomotory activities have not been examined.

## Muscle size

Once the electrical signal reaches the muscle, the primary determinant of muscle strength is its size. This can be measured in several ways, including a “gold standard” measurement of 3D volume of individual muscles using MRI or CT, which has not been completed in individuals with OI. Clinically, particularly in adults, the assessment of lean mass via 2D projections from DXA scans is used. Studies in OI have used DXA, and 2D measurements of cross-sectional area (CSA) via peripheral quantitative CT (pQCT).

Previous pQCT studies have shown 6%-8% lower calf and forearm muscle CSA in children and adolescents with OI type I compared to age and sex matched controls,^7^^,^^28^^,^^55^ with 12% lower values in type I adults.^5^ Similar findings were observed in Col1a1^Jrt/+^, *oim*/*oim* and homozygous *G610C* mice.^12^^,^^13^^,^^36^^,^^56^ The comparative rarity of other, more severe OI types, may have contributed to the limited information available on muscle in these groups, evidenced by the smaller number of participants from rarer OI types in studies, where multiple types have been assessed.^5^^,^^55^ In addition, due to the need to exclude participants with metal implants or where limbs are too short to be scanned, the few studies which have been performed may not be as representative of these populations. For example, in our previous study in adults,^5^ 100% of participants with type I OI participated in pQCT and DXA imaging, but while DXA participation in type III was 100% only 40% could complete pQCT tibia measures. Similarly, differential rates of participation were evident across muscle function tests, including hand grip (type I 98% type II 100%), shoulder abductor strength (type I 100%, type III 50%), and wall sit (type I 91%, type III 25%). In children, forearm CSA deficits in those with type I and type IV (both 8% less than controls) appeared smaller than type III (14%), although small group size did not permit well-powered inter-group comparisons.^55^ These differences in CSA are independent of concurrent differences in limb length in more severe OI types. These contribute to far greater adult deficits in appendicular lean mass (49% in type III, 43% in type IV and V) than in those with type I (12%),^5^ which are all also independent of body size and physical activity.^6^ In contrast, in children with OI type I (height Z-score −1.1) and type IV (−1.2) of similar stature, deficits in whole body lean mass were equivalent (SDS −2.5 and −2.8, respectively).^57^ Similar muscle size deficits independent of body size have also been observed in the *oim* mouse model.^13^

Lower wet weight of multiple muscles has been observed in mouse models, with more pronounced deficits in severe *oim* mice^14^ compared to milder heterozygous *G610C.*^36^ Muscle size is the product of myofiber and mitochondrial (non-contractile) muscle CSA, fiber number and fiber length therefore differences in one or more these properties will underly reduced muscle size. Hypoplasia (reduced fiber number) has been observed in Col1a1^Jrt/+^ diaphragm muscle,^12^ and in soleus but not extensor digitorum longus of male but not *oim* mice.^58^ Myofibrillar CSA was similar in Col1a1^Jrt/+^ mice diaphragm^12^ and 4 hindlimb muscles across *G610C* sexes (with the exception of smaller gastrocnemius CSA in females),^36^ but mitochondrial CSA and fiber length remain unexplored.

Myokines secreted by muscle have important paracrine and endocrine effects, including those on metabolism, cardiovascular, and mental health,^59^ hence smaller muscle size in OI likely has non-mechanical effects on these systems. Animal OI models suggest that both myokines and bone-secreted osteokines differ in OI.^60^ Adults with OI have higher levels of the osteokine osteocalcin,^61^^,^^62^ although little is known from human studies about how this might impact on muscle. More broadly, beyond simple association studies (in which directionality and causality cannot be attributed), little is known about the consequences of the bi-directional biochemical crosstalk between muscle and bone in humans. Another osteokine lipocalin 2 (LCN2) was positively associated with total and appendicular lean mass but not grip strength in 204 children with different OI types^63^ However, these univariate associations may be confounded by negative associations with age, BMI, and certain OI types and genotypes, which were not adjusted for in analyses.

## Muscle quality

Muscle function is also dependent upon the quality as well as quantity of muscle tissue. Muscle quality is the measure of muscle strength or power per unit of muscle mass. It is influenced by the composition, architecture, and metabolism of skeletal muscle, as well as neural activation.^64^ To the best of our knowledge, histological examination of muscle via biopsy in humans with OI is limited to a couple of individuals.^8^^,^^65^ The first is from a 2-yr-old presenting with muscle weakness and subsequently diagnosed with OI type IV, with a muscle biopsy revealing enlarged mitochondria but no other morphological differences.^8^ The second, from a boy aged 11 yr diagnosed with a rare non-collagenous OI type XVII, also showed prominent mitochondria, excess lipids and some variation in fiber size.^65^ Therefore, in the absence of direct assessment of cell and molecular changes in muscle in humans with OI, muscle density measured by CT is commonly used. Muscle density is negatively correlated with muscle lipid content^66^ and axonal degeneration^67^ and positively associated with muscle function^68^ and locomotory function^69^ independent of muscle size. Therefore, it is considered a proxy for muscle quality. Adults with OI type I have 2%-3% lower muscle density at the calf muscle than age and sex-matched controls,^5^ although conflicting findings have been observed in children with similar density at the calf but 14%-19% lower trunk muscle values in a cohort containing 75% OI type I.^7^^,^^28^^,^^70^

Mouse models have offered more insights into possible mechanisms underlying functional deficits. Lower specific (adjusted for muscle size) force, indicating reduced muscle quality or fiber type change, has been reported in *oim* hindlimb^13^ and *Col1a1^Jrt/+^* diaphragm muscle,^12^ with no difference in milder *G610C* mice. Type I collagen is found primarily within the epimysium,^71^ which covers the belly of skeletal muscles. The epimysium has a key role in intramuscular transmission of force,^72^ hence altered collagen in OI may directly affect force generation. Fibrillar collagen content, which contributes to muscle stiffness and strength, is reduced.^13^ There are mixed findings with regards to fiber type difference, which would have differential effects on power and endurance performance. Null results (*oim* heterozygous and homozygous),^13^ and lower type I and increased type IIa (*oim* homozygous)^58^ being reported in soleus, with increased IIa and reduced IIx fibers (*Col1a1^Jrt/+^*) in diaphragm muscle.^12^

Reduced mitochondrial function was observed in severe but not mild OI animal models,^14^^,^^58^^,^^73^ with increased energy expenditure but unaltered substrate utilisation^58^ and muscle glycogen content^14^ in *oim*. These metabolic and mitochondrial changes may underly functional deficits including lower power and resistance to fatigue in humans. Gremminger et al. identified mitochondrial and metabolic changes in *+/G610C* mice, including reduced state 3 mitochondrial respiration, increased citrate synthase activity, elevated Parkin and p62 protein levels, and a higher respiratory quotient.^73^ These alterations may reflect compensatory mechanisms that prevent muscle weakness in these mice compared to severe OI models. Transcriptomic analysis of gastrocnemius muscle in 2 severe OI models (homozygous *oim* and heterozygous *Col1a1^Jrt/+^*) identified 27 shared differentially expressed genes, with upregulated genes related to lipid metabolism and extracellular matrix components, and downregulated genes associated with muscle contraction pathways, particularly those coding for slow-twitch type I fibers.^74^ Mss51, a mitochondrial metabolic stress-inducible factor, was also downregulated. The authors concluded that these findings suggest that muscle disturbances in severe OI models are multi-factorial in origin and resemble a mild form of muscular dystrophy.

## Tendon in OI

Once skeletal muscle contracts, the forces produced are transmitted through a pliable tendon to bone to create joint torques. Like muscle and bone, tendon’s primary functional capacity (its stiffness) changes substantially with growth and aging^75^^,^^76^ and physical activity, and a more pliable tendon is associated with a lower rate of force development.^76^ As highlighted earlier, tendons are predominately made of collagen type I accounting for 70%-80% of their dry weight. In Crtap, *oim* and *Col1a1^Jrt/+^* mice tendons had lower CSA, stiffness, and toughness although material level mechanical properties were not significantly different and differences in underlying collagen structure were mutation-dependent.^31^^,^^77–80^ It is currently unclear to what extent these differences reflect an underlying collagen or other defect, or reduced loading due to muscle weakness and physical inactivity. No assessment of tendon structure or function has been reported in humans with OI. Injuries to tendons and ligaments (also ~80% collagen type I by dry weight^81^) are common in OI^82^ although incidence and risk factors have not been objectively characterized.

## Extramuscular factors contributing to muscle deficits in OI

Lifestyle factors and altered function of, for example, cardiovascular, respiratory, and metabolic systems, may contribute to impaired muscle function in OI. Two key determinants of muscle size and function are physical activity and diet. Individuals with OI may be less active, particularly in moderate-vigorous activities, due to pain and fear of injury.^83^^,^^84^ In line with this, step count and moderate-vigorous physical activity assessed by accelerometry were lower in adults with OI type I than controls.^6^ While no association was observed in children and adolescents,^85^ this may relate to the small study size (14 in each group) given substantial inter-individual variation in these measures. These assessments do not consider periods of physical inactivity following fracture or surgery, which may be considerable. Muscle size and function changes rapidly in response to disuse, with 10% lower force and 5% reduced volume in the calf muscle in healthy adults following 14 d of bed rest.^86^ In animal studies, lower physical activity has been observed in more severe but not milder models.^14^^,^^31^^,^^36^^,^^53^^,^^73^ To date, the relationship between physical activity and muscle in OI remains unexplored. Data on nutritional intake are very limited^87^^,^^88^ and do not take into account altered metabolism observed in OI in children^63^^,^^89^ and *oim* mice.^58^

OI is associated with cardiac abnormalities,^90^ including reduced cardiac muscle function in human and animal studies,^91–93^ with consequences for gas and nutrient transport and in turn muscle function. Impaired ventilatory function, including reduced respiratory muscle strength,^94^ is also evident in children with OI^9^ as well as adults with more severe types,^95–97^ and has also been reported in multiple animal models of OI.^98^^,^^99^ These deficits can be exacerbated in the severe OI types by rib cage deformities affecting respiratory muscle function^97^ and in turn affecting functional capacity of other skeletal muscles. More broadly, the presence of skeletal deformities is associated with poorer muscle function in individuals with the condition.^11^ Altered metabolism in children with OI^89^ potentially mediated by osteocalcin^100^ through which resting oxygen consumption and energy expenditure are increased, may also impact energetics during physical activity, limiting functional capacity. Metabolic disturbances were observed in *Col1a1^Jrt/+^* mice including a hypermetabolic state with elevated oxygen consumption and energy expenditure, potentially worsening OI pathology.^100^ Given that muscle has a key role in whole-body metabolism, and is a primary determinant of inter-individual variation in energy expenditure,^101^ it is likely that there are reciprocal effects between muscle and metabolism in OI. We have also shown that higher levels of pain and fatigue are associated with impaired physical function in OI.^27^

## Epidemiology of OI muscle deficits

The epidemiology of muscle deficits in OI remains little explored, likely due to the rarity of the condition and the challenges of muscle assessment in OI detailed below.

Our clearest understanding relates to muscle function in different OI types, for which as described above there is clear evidence of greater deficits in multiple components of muscle function in more severe OI types.^5^^,^^55^ It is also important to note that there is wide variation in muscle function in all OI types (Figure 1). This may relate to the underlying mutation; previous studies did not find an independent association with haploinsufficiency or frameshift vs other mutations although analyses involved multiple regression within relatively small cohort so may have been underpowered.^7^^,^^28^ There are no studies examining muscle function in non-collagenous mutation OI types, only one case report of muscle weakness,^65^ and only a single mouse study which found reduced physical activity, impaired motor control and muscle weakness in a cartilage-associated protein (CRTAP) knockout model.^31^

To the best of our knowledge, nothing is known about whether deficits in muscle function relative to controls differ according to sex. Also, age trajectories of muscle function across childhood and adulthood, which might indicate key periods for diagnosis and intervention on muscle weakness, are lacking. The only studies to examine muscle function longitudinally used a subjective manual muscle grading system^102^^,^^103^; while values did not change with time, no comparison to controls was made. As described above, results from separate studies in children and adults suggest that deficits in some aspects of function may be more impaired in adults. However, the lack of data using a consistent methodology across age groups limits our current understanding.

Thirty-three percent of children with type I OI demonstrated jump power above that of the reference data mean,^28^ suggesting that diagnostic testing is required to identify those individuals with a muscle deficit. This raises an important question about the most relevant aspects of function for different types. For individuals with OI type I, of whom over 80% can walk 500 m without assistance, muscle function limitations are very different to those with OI type III of whom more than 40% require a wheelchair to travel 5 m.^32^ As a result, we need to consider which aspect(s) of muscle function are most relevant to quality of life, physical function, and clinical risk in each group, and which are feasible. Due to issues, such as pain, skeletal deformity, fatigue, and fear of injury, many individuals with OI (particularly severe types) may not be able to complete common muscle function assessments. While 100% of adults with OI type III completed hand grip, only 5/12 (42%) could complete a chair rise test and none produced a valid jump or hop.^5^ After selection of the most relevant assessments, type-specific thresholds for impaired function must be calculated against which risk factors and interventional strategies can be developed.

## Non-pharmacological and pharmacological interventions for alleviating muscle dysfunction in OI

A small number of interventional studies have been performed to address deficits in muscle function in OI in children, but to date there are no studies in adults. An intensive 6-mo rehabilitation program incorporating whole-body vibration training led to increased lean mass and improved mobility, although no control arm was examined.^104^ In a 5-mo randomized control trial of a more pragmatic 3×3 min daily vibration training, lean mass increased relative to controls, but this was not accompanied by significant increases in muscle function as assessed by dynamometry.^105^ A 12-wk training program increased functional capacity by 10% and muscle strength by 12% in children with OI types I and IV.^40^ The training included 30 sessions of 45 min incorporating aerobic training at 60%-80% of the maximum heart rate (although precise modality—running, cycling, etc., is unknown), strength exercises and free play. In contrast to these findings in less severe OI types, male *oim* mice displayed lower muscle mass and function following running and swimming exercise compared to controls.^14^ A lack of empirical evidence limits clinical guidance for muscle health and function in OI, although an expert panel of physiotherapists, occupational therapists, and medical doctors across 5 continents have published a 17-point consensus statement on physical rehabilitation in children and adolescents.^106^

Genetic and pharmacological inhibition of myostatin, a negative regulator of muscle mass, increases muscle mass in OI animal models.^107^ Myostatin inhibitors have been developed and applied in human adult trials^108^ but have not been applied in OI. Anti-resorptive bisphosphonate treatment is commonly used to address low bone density in individuals with OI. There is conflicting evidence as to whether bisphosphonates also have a beneficial effect on muscle function, with positive findings in a three-year longitudinal study of intravenous pamindronate involving comparison to historic controls^109^ contrasting with null findings in shorter-term and smaller-scale controlled trials of oral pamidronate.^110^^,^^111^ Adults with OI treated with bisphosphonates in childhood have higher physical function than those treated in adulthood.^112^ In the absence of effects on muscle mass, it may be that reduced bone pain following treatment leads to improved function, or alternatively that lower resorption limits the release of factors such as TGF-β.^113^ Excessive TGF-β signaling has been implicated as a pathogenic mechanism in OI, and anti-TGF-β treatments improve the muscle mass deficit observed in OI mouse models.^114–116^

## Conclusions

Taken together, current data show pronounced deficits in multiple components of muscle function in both children and adults with OI of all types. These have important clinical and functional implications for aspects such as skeletal health, fall and fracture risk, mobility, and the ability to carry out activities of daily living. These deficits appear multifactorial in origin, with contributions from impaired sensory, respiratory, and metabolic function in addition to low muscle mass and impaired muscle quality. There are several important gaps in knowledge in our understanding of muscle in OI which limit our ability to develop risk stratification and interventional strategies: (1) with patient involvement, consensus around the most clinically and functionally relevant aspects of muscle function and health needs to be derived, so that research is focused on those components with the greatest impact on health and quality which likely differs by OI type. This will facilitate (2) deeper characterization of multiple clinically-and functionally-relevant components of muscle function across individuals of different ages with different OI types, including behavioral and other environmental exposures. This is essential in order to identify the impact of OI on muscle at different life stages and to best target interventions. Finally, as priority, (3) the limited amount of information on the fundamental mechanisms underlying these deficits also needs to be addressed. By using detailed techniques established for human clinical studies such as muscle biopsies, motion capture and imaging using modalities, such as MRI, we can better describe the multi-factorial basis of muscle deficits in OI and thereafter develop novel, effective therapeutic strategies. Investigating these unresolved aspects should be a priority for basic and clinical researchers in the field.

## Data Availability

The data supporting the findings of this review are available within the articles cited in the references.
